# Supplementary material for: Surface Structure Engineering of PdAg Alloys with Boosted CO2 Electrochemical Reduction Performance
Source: Nanomaterials (Basel). 2022 Nov 1;12(21):3860. doi: 10.3390/nano12213860 (PMC9657775; doi:10.3390/nano12213860)
Supplement: Supplementary file 1 [file nanomaterials-12-03860-s001.zip › nanomaterials-1998000-supplementary.pdf]

Supplementary Materials

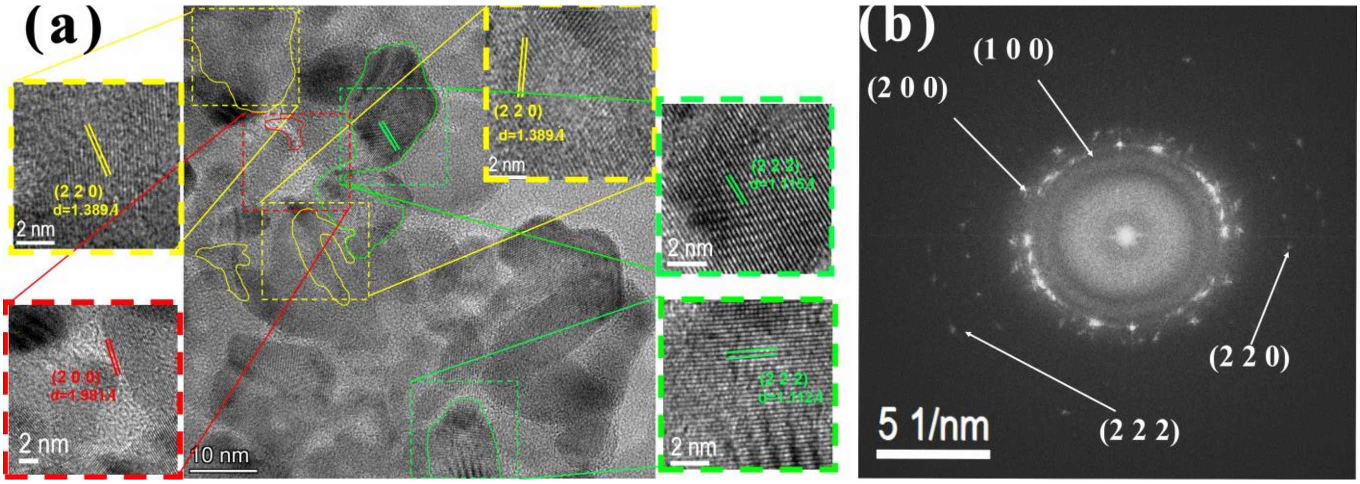

Figure S1. (**a**) high-resolution TEM images of Pd_3Ag; (**b**) Selective Area Electron Diffraction (SAED) of Pd_3Ag.

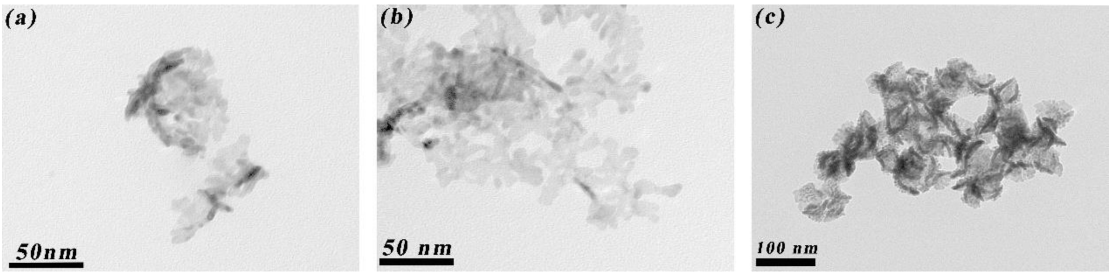

Figure S2. TEM images of (**a**) Pd/Ag=2:1; (**b**) Pd/Ag=1:1; (**c**) pure Pd.

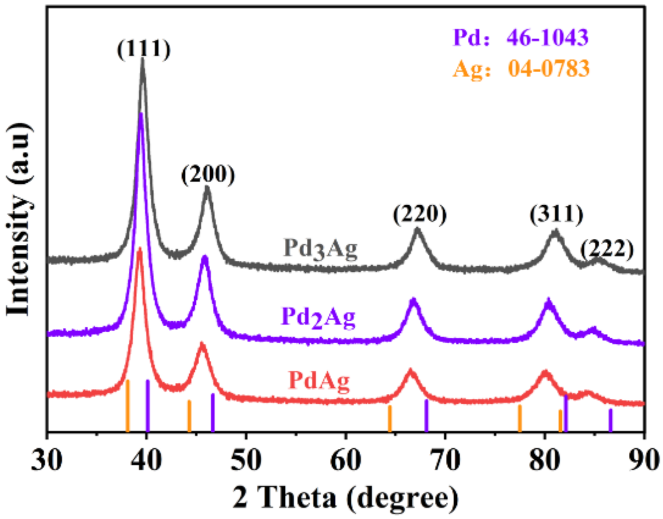

Figure S3. XRD patterns of Pd_3Ag, Pd_2Ag, and PdAg.

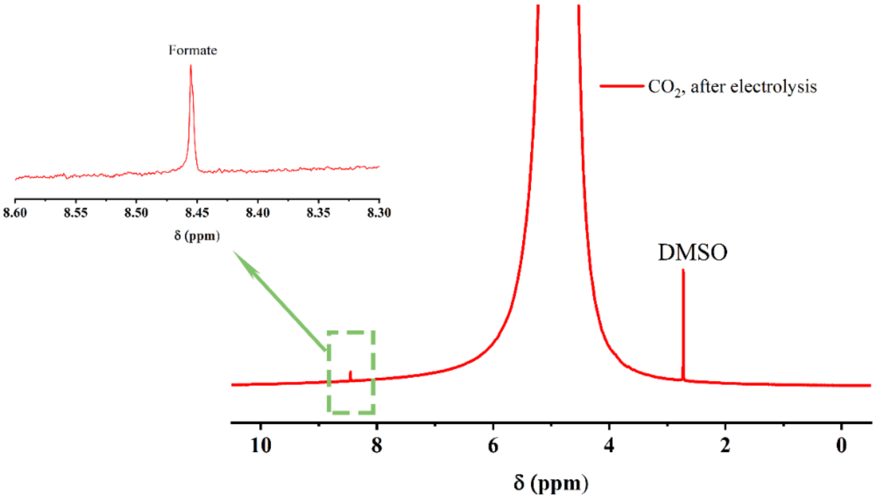

Figure S4. ^1H NMR spectra of the liquid conversion products for Pd_3Ag alloy after electrolysis for 1 h at -0.2 V(vs RHE).

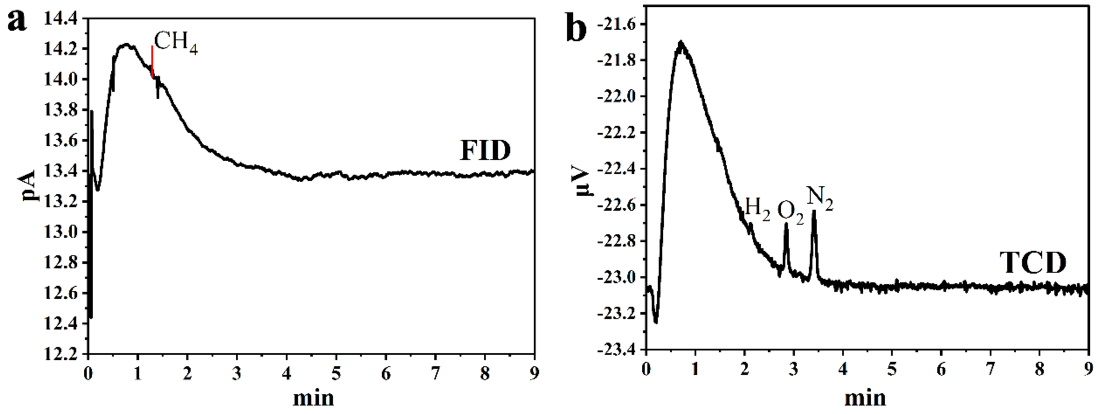

Figure S5. GC spectra of the gas conversion products detected via (**a**) Thermal Conductivity Detector (TCD) and (**b**) Flame Ionization Detector (FID) for Pd_3Ag alloy after electrolysis for 1 h at -0.2 V (vs RHE).

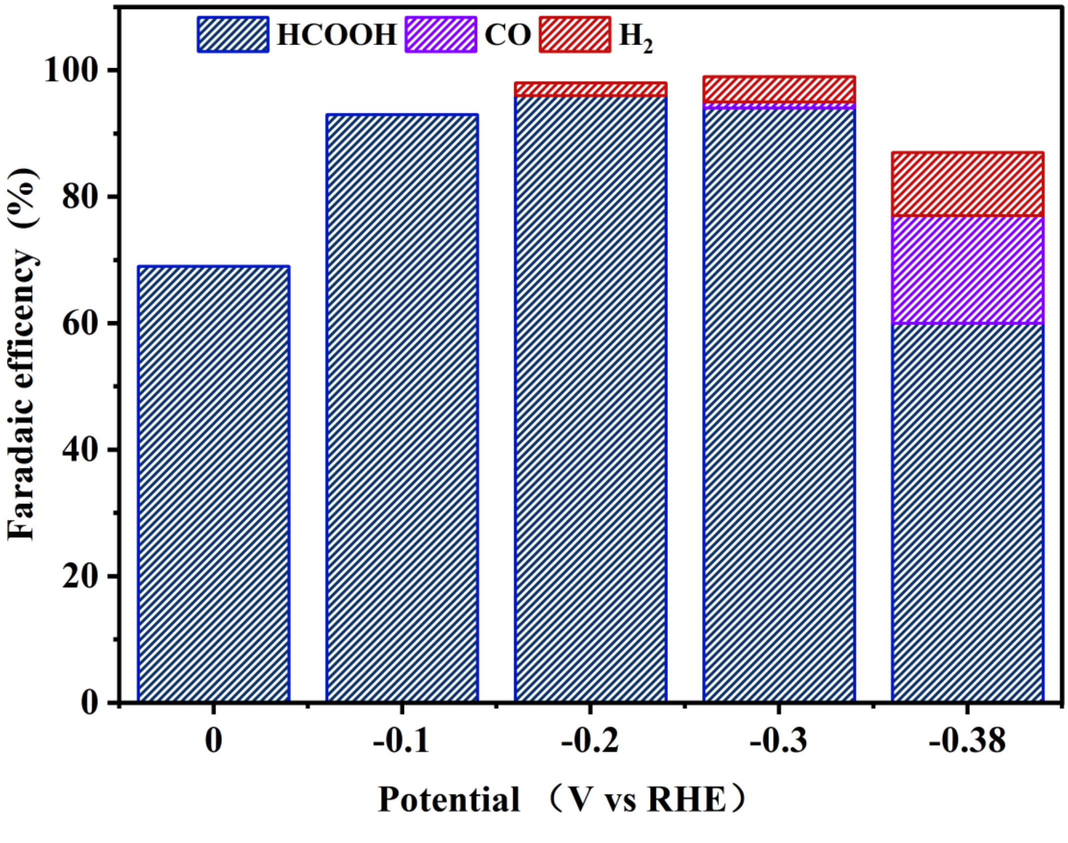

Figure S6. Potential-dependent FE for CRR products and partial current density.

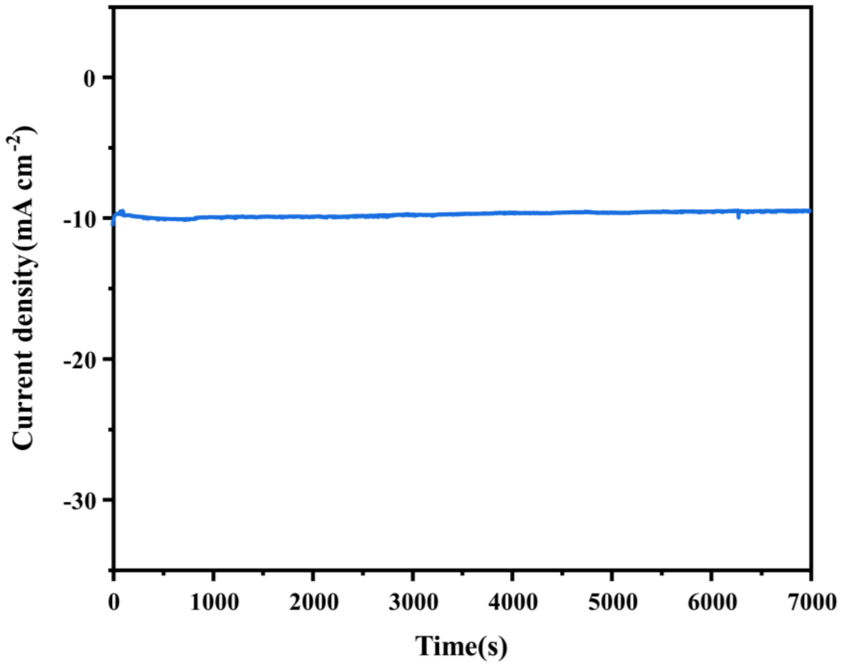

Figure S7. Chronoamperometric curves for Pd_3Ag at -0.2 V (vs RHE) in CRR.

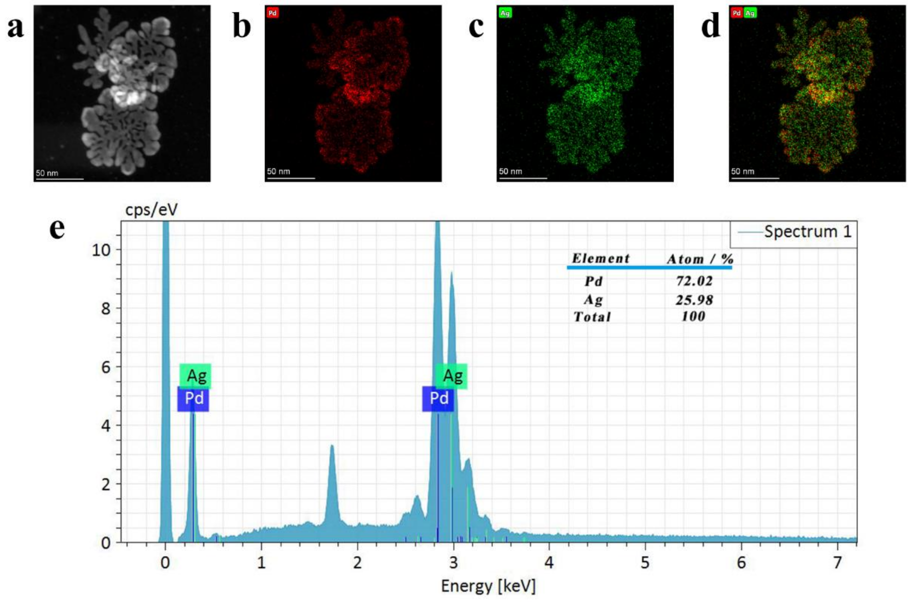

Figure S8. Structural characterizations of bimetallic Pd_3Ag after long-term durability test. (**a**) HADDF-STEM-EDS mapping of Pd and Ag in the selected area. Elemental mapping of (**b**)Pd, (**c**) Ag, and (**d**) a mixed pattern of Pd and Ag. (**e**) The EDX spectra of the Pd_3Ag after a long-term durability test.

Table S1. Comparison of different catalysts in electrochemical CRR for HCOOH formation.

catalysts	product	FE	Overpotential/ V (vs. reference hydrogen electrode)		Refernece No
**Pd_3Ag**	HCOOH	96%	-0.2		**this work**
Pd_4Ag nanowires	HCOOH	90%	-0.3		1
$Pd_{70}Pt_{30}$/C nanoparticles	HCOOH	88%	-0.4		2
Pd nanoparticle films	HCOOH	50~60%	-0.4		3
Nanoporous Ag-Sn	HCOOH	85%	-0.8		4
Ag-Sn Bimetallic	HCOOH	80%	-0.8		5
Pd–B/C	HCOOH	70%	-0.5		6
4,4′,4″,4‴(1,4phenylenebis(pyridine-4,2,6-triyl))tetrabenzoic acid	HCOOH	90.5%	−1.8		7

References

1. Han, N.; Sun, M.; Zhou, Y.; Xu, J.; Cheng, C.; Zhou, R.; Zhang, L.; Luo, J.; Huang, B.; Li, Y., Alloyed Palladium-Silver Nanowires Enabling Ultrastable Carbon Dioxide Reduction to Formate. *Adv. Mater.* **2021,** *33* (4), 2005821.
2. Kortlever, R.; Balemans, C.; Kwon, Y.; Koper, M. T. M., Electrochemical CO_2 reduction to formic acid on a Pd-based formic acid oxidation catalyst. *Catal. Today* **2015,** *244*, 58-62.
3. Zhou, F. L.; Li, H. T.; Fournier, M.; MacFarlane, D. R., Electrocatalytic CO_2 Reduction to Formate at Low Overpotentials on Electrodeposited Pd Films: Stabilized Performance by Suppression of CO Formation. *Chemsuschem* **2017,** *10* (7), 1509-1516.
4. Wang, X.; Xiao, W.; Zhang, J.; Wang, Z.; Jin, X., Nanoporous Ag-Sn derived from codeposited AgCl-SnO2 for the electrocatalytic reduction of CO_2 with high formate selectivity. *Electrochem. Commun.* **2019,** *102*, 52-56.
5. Luc, W.; Collins, C.; Wang, S. W.; Xin, H. L.; He, K.; Kang, Y. J.; Jiao, F., Ag-Sn Bimetallic Catalyst with a Core-Shell Structure for CO_2 Reduction. *J. Am. Chem. Soc.* **2017,** *139* (5), 1885-1893.
6. Jiang, B.; Zhang, X. G.; Jiang, K.; Wu, D. Y.; Cai, W. B., Boosting Formate Production in Electrocatalytic CO_2 Reduction over Wide Potential Window on Pd Surfaces. *J. Am. Chem. Soc.* **2018,** *140* (8), 2880-2889.
7. Kang, X.; Li, L.; Sheveleva, A.; Han, X.; Li, J.; Liu, L.; Tuna, F.; McInnes, E. J. L.; Han, B.; Yang, S.; Schroder, M., Electro-reduction of carbon dioxide at low over-potential at a metal-organic framework decorated cathode. *Nat Commun* **2020,** *11* (1), 5464.
